# Regulatory cells and the effect of cancer immunotherapy

**DOI:** 10.1186/s12943-023-01714-0

**Published:** 2023-02-04

**Authors:** María Iglesias-Escudero, Noelia Arias-González, Eva Martínez-Cáceres

**Affiliations:** 1 Immunology Division, LCMN, Germans Trias i Pujol University Hospital and Research Institute, Campus Can Ruti, Badalona, Spain; 2 Department of Cellular Biology, Physiology and Immunology, Universitat Autònoma de Barcelona, Cerdanyola del Vallès, Spain

**Keywords:** Tumor, Immunotherapy, Regulatory cells, Checkpoint inhibitors, MDSCs, Tregs, TAMs

## Abstract

Several mechanisms and cell types are involved in the regulation of the immune response. These include mostly regulatory T cells (Tregs), regulatory macrophages (Mregs), myeloid suppressor cells (MDSCs) and other regulatory cell types such as tolerogenic dendritic cells (tolDCs), regulatory B cells (Bregs), and mesenchymal stem cells (MSCs). These regulatory cells, known for their ability to suppress immune responses, can also suppress the anti-tumor immune response. The infiltration of many regulatory cells into tumor tissues is therefore associated with a poor prognosis. There is growing evidence that elimination of Tregs enhances anti-tumor immune responses. However, the systemic depletion of Treg cells can simultaneously cause deleterious autoimmunity. Furthermore, since regulatory cells are characterized by their high level of expression of immune checkpoints, it is also expected that immune checkpoint inhibitors perform part of their function by blocking these molecules and enhancing the immune response. This indicates that immunotherapy does not only act by activating specific effector T cells but can also directly or indirectly attenuate the suppressive activity of regulatory cells in tumor tissues. This review aims to draw together our current knowledge about the effect of immunotherapy on the various types of regulatory cells, and how these effects may be beneficial in the response to immunotherapy.

## Introduction

Tumors can modify the microenvironment by releasing extracellular molecules, inducing tumor angiogenesis and promoting peripheral immune tolerance, while the immune cells in the microenvironment can affect the growth and evolution of cancerous cells. Increasing amounts of T CD3 + , cytotoxic CD8 + and memory CD45RO + T cells are associated with greater disease-free survival and overall survival (OS) in most studies [1–3]. Histological analysis of tumors has highlighted the importance of immunological infiltrates, including macrophages, dendritic cells, polymorphonuclear cells, natural killer (NK) cells, B cells and T cells, and revealed a wide diversity of these among patients [4]. Although the extent of the immune infiltrate can be a good prognostic indicator in some cancers, the anti-tumor response is clearly insufficient to prevent disease progression. In inflamed tumors, negative immune regulatory factors tend to be dominant due to the chronic nature of the immune infiltrate. For instance, in a study aimed at identifying biomarkers associated with clinical outcome in melanoma patients, the decrease in FOXP3 + /regulatory T cells (Tregs) was associated with better clinical responses in the group treated with ipilimumab [5].

Therefore, patients with tumors containing infiltrates could be induced to respond to immunotherapy if immune cells within the microenvironment are reactivated. On the other hand, the presence of regulatory cells such as Tregs, MDSCs (myeloid-derived suppressor cells) and TAMs (tumor-associated macrophages) has generally been associated with a poor clinical prognosis. The actions on the vast majority of these components that have been reviewed here are not as advanced in head and neck cancer (HNC) as in other neoplasms such as melanoma.

Although therapy based on immune checkpoint blockade has achieved outstanding results in terms of OS [6], a high percentage of patients still show intrinsic resistance [7]. Some features are required to achieve a successful response to programmed cell death protein 1 (PD-1) blockade, such as a high frequency of tumor neoantigens, the amount of infiltrating effector T cells, a high level of expression of PD-L1, or an IFN-related gene signature [8]. Due to their potent suppressive activities against effector lymphocytes and their abundance in the tumor microenvironment, immunosuppressive cells act as a major barrier to cancer immunotherapy. A variety of therapeutic approaches directed towards immunosuppressive cells are actively being tested in preclinical and clinical studies [9–14].

**Fig. 1:**
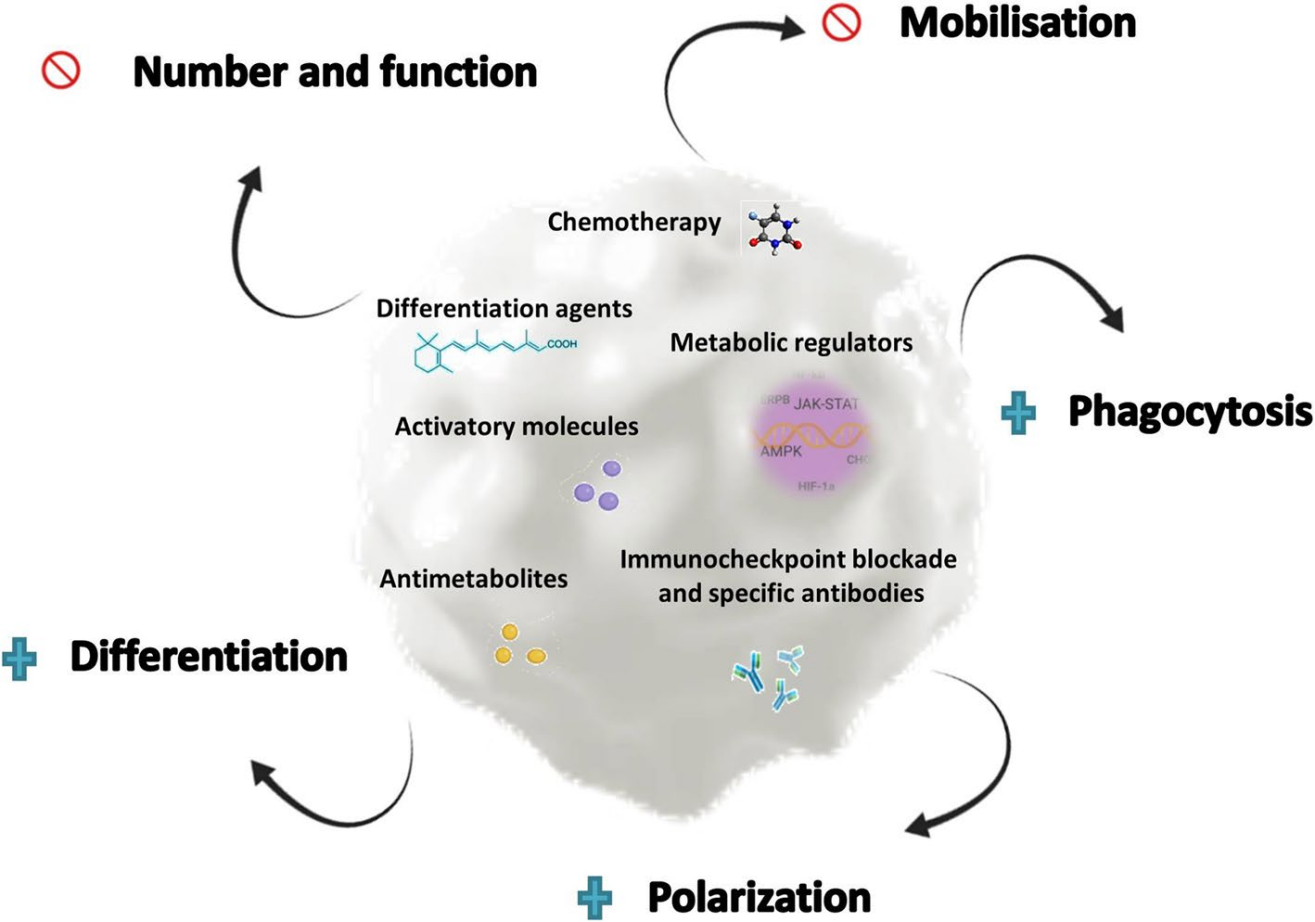
Strategies to reduce regulatory cells in cancer patients. Several strategies alone or combining the targeting of different biochemical pathways have been reported to modulate regulatory cells in cancer patients. Some approaches aim to reduce the frequency, function and extent of blocking mobilization, while others focus on enabling phagocytosis, polarization, or potentiating differentiation. Blue crosses indicate potentiation/increase; forbidden sign indicates blockade/reduction

Since regulatory cells are characterized by their high level of expression of immune checkpoints, it is also expected that immune checkpoint inhibitors perform part of their function by blocking these molecules and enhancing the immune responses. This suggests that immunotherapy does not only act by activating specific effector T cells but also can directly or indirectly attenuate the suppressive activity of regulatory cells in tumor tissues. The aim of this review is to draw together the current knowledge about the effect of immunotherapy on the various types of regulatory cells, and how these effects may be beneficial in the response to immunotherapy (Fig. 1).

## Tregs

**Fig. 2:**
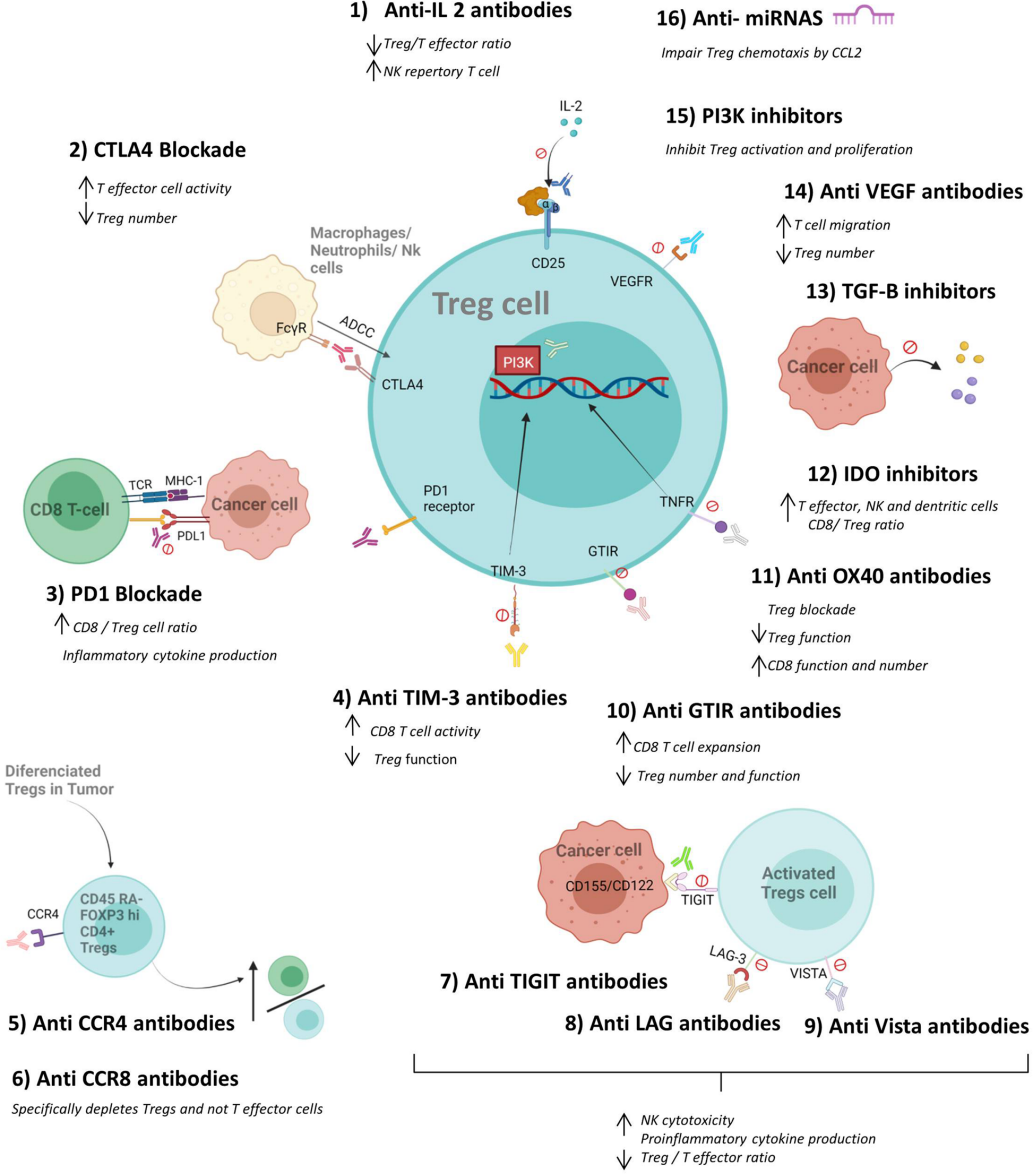
Effect of immunotherapy on Tregs. Several ICIs and other treatments can directly affect the number and function of regulatory T cells consequently, improving anti-tumoral function and preventing tumor growth. Some treatments act by reducing the number of Tregs (by antibody dependent cellular citotoxicity, ADCC) while others affect their suppressive activity or positively modulate the functions of CD8 T, NK cells and dendritic cells. The modulation towards pro-inflammatory cytokines and the increase in the CD4 + effector/Treg ratio are other mechanisms by which immunotherapy can potentiate the immune response within the tumor microenvironment. Created with Biorender.com

The activities of Tregs are the most widely studied of the mechanisms regulating the immune response. They were discovered several decades ago [15] and are known to have a strategic role in the maintenance of immune homeostasis [16]. Their function has been closely linked to the development of diverse pathologies, including autoimmunity [17] and cancer [18]. As Tregs are known to express many immune checkpoint inhibitor (ICI) targets, the effect of these immunotherapies could alter Treg numbers and function. For this reason, a comprehensive understanding of their action in cancer settings could help increase the efficacy and reduce the incidence of immune-related adverse effects after ICI treatment (Fig. 2).

In the clinical setting, the use of daclizumab (a humanized neutralizing monoclonal antibody against the α-chain of the interleukin-2 receptor) in patients with metastatic melanoma resulted in depletion of Tregs and effector T cells, so that augmented T cell responses in the tumor are not induced [19]. In contrast, in a study of breast cancer patients, administration of daclizumab followed by vaccination with multiple tumor-associated peptides reduced the frequency of Tregs and gave rise to a durable, stable disease with little progression [20–22]. Denileukin diftitox (a ligand–toxin fusion protein that consists of full-length IL-2, and which has been approved for the treatment of CD25 + cutaneous T-cell leukemia and lymphoma) was used for lung, ovarian and breast cancer, demonstrating that a single dose reduced the prevalence and absolute numbers of peripheral Tregs and increased effector T-cell activation in all patients [23, 24]. Considering all the previous results, it was inferred that therapeutic success may depend on the phase and state of the anti-tumor immune responses [25].

Several reports have suggested that the effect of ipilimumab (anti CTLA-4, human antibody IgG1 isotype) on Tregs is mediated only through antibody-dependent cell-mediated cytotoxicity, by which their suppressive functions are maintained [26–28]. On the other hand, tremelimumab (anti CTLA-4 antibody IgG2 isotype) has been shown to be able to replenish effector and memory CD4 + and CD8 + T-cell numbers by suppressing Treg activity without influencing the proportion of Tregs [29]. A recent study showed that ipilimumab and tremelimumab both increase infiltration of intratumoral CD4 + and CD8 + cells without depleting FOXP3 + cells in human tumors, suggesting that their efficacy could be enhanced by modifying the Fc portions of the monoclonal antibodies (mAbs) to enhance Fc-mediated depletion of intratumoral regulatory T cells [30]. Also, it has been described that a combined treatment with anti B7x may help to overcome the B7x-mediated resistance to anti-CTLA-4 [31].

However, the mechanism underlying the action of these antibodies is unknown. The discrepancies could be explained by the fact that the number of peripheral Tregs depends on the genetic background of the patients and the time since immunotherapy that the analysis is performed.

PD1 blockade can induce CD8 + T-cell proliferation and cytokine production, thereby negatively regulating Treg numbers by increasing the CD8 + Teff-to-Treg ratio [32]. The suppressive capacity of effector Tregs was enhanced in some advanced gastric cancer patients treated with PD1 blockade and was associated with rapid cancer progression [33]. This pattern has also been described by other authors [34]. Considering these findings together, the reports suggest that a combination of anti-CTLA4-depleting Tregs with PD1 blockade could be used to enhance CD8 activation and Treg depletion. It would also be interesting to study the effects of the timing and duration of such a Treg-targeting antibody treatment, to determine whether it is critical for the differential control of Treg and effector T cells, in other words, whether the longer the treatment, the more likely effector T cells are to be depleted, thereby hindering the generation of effective tumor immunity [18, 35]. The anti-CCR4 antibody, mogamulizumab, has demonstrated an effective reduction in the frequency of effector Tregs that selectively augment the induction of tumor antigen-specific CD4 + and CD8 + T cells in vivo [36, 37].

Moreover, anti CCR8 monoclonal antibodies were shown to cure tumors in mice by selectively depleting tumor Tregs and increasing CD8 + effector T cells [38]. *Van Damme *et al. also demonstrated that anti-CCR8 antibodies had antitumor effects and were seen to display synergistic antitumor effects combined with anti-PD-1 mAbs [39]. More recent studies describe the use of microRNAs -15a/16–1 to regulate immunosuppression in hepatocellular carcinoma by reducing CCL22 binding to C–C chemokine receptor type 4 on Tregs [40].

New therapeutic strategies are being developed to target the major co-inhibitory and co-stimulatory molecules of Tregs. Some TIGIT monoclonal antibodies (BMS-986207) are under clinical trial in combination with nivolumab for the treatment of advanced solid tumors (NCT02913313) and as monotherapy or in combination with atezolizumab (anti PD-1 antibody) (MTIG7192A) [41–43]. Studies of patients with melanoma show that LAG-3 + Tregs selectively expand in PBMCs and TILs, bestowing potent suppressive activity on them in a cell-to-cell, contact-dependent manner [42].

Other approaches, such as blockade of TIM-3 [44], V-domain Ig suppressor of T cell activation (VISTA) [45, 46], treatment with glucocorticoid-induced TNFR-related (GITR) protein agonistic antibody [47, 48], anti-OX40 antibodies [49–51], IDO-1 inhibitors [52, 53], TGF-β inhibitors [54]VEGF-targeting therapy receptor 2 (VEGFR2) [55, 56], PI3K inhibitor and HSP inhibitor (phosphoinositide 3-kinase (PI3K) pathway, or heat shock protein (HSP) [57], have also yielded promising results. Some drugs in combination with immune checkpoint inhibitors are currently the subject of ongoing clinical trials for cancer therapy [58].

Others approaches such as vaccines, nanodrugs [59], the generation of chimeric antigen receptor (CAR-)T cells, [60] or directly targeting FOXP3 in Tregs with an antisense oligonucleotide are also under investigation with the aim of reprogramming Tregs [61].

## MDSCs

**Fig. 3:**
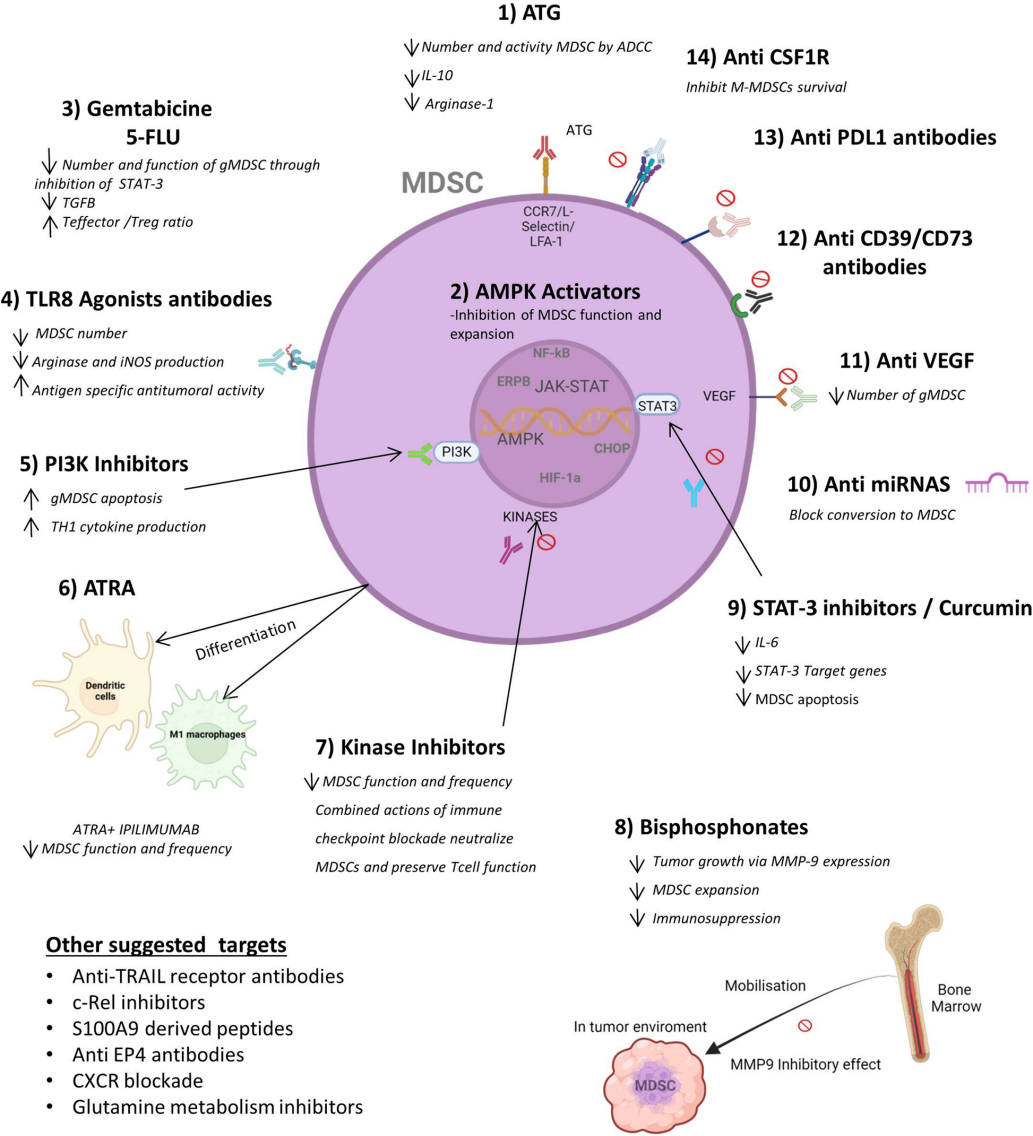
Effect of immunotherapy on MDSCs. The figure depicts different treatments that can modulate MDSC expansion and function. The drugs affect the frequency of MDSCs, reduce MDSC suppressive activity, improve CD8 T cell function, and modulate the release of cytokines from the tumor. A combination of these therapies may have a synergistic effect that promotes better outcomes. Created with Biorender.com

MDSCs are recognized as one of the major cell components in the tumor microenvironment, where they promote tumor growth by exerting their immunosuppressive functions. MDSCs have emerged as major regulators of immune responses in cancer and key targets for treating cancer [62] (Fig. 3).

Suk Lee et al. reported that anti-thymocyte globulin (ATG)-treated mice showed a reduction in MDSCs frequency and function, suggesting that ATG could be used to suppress MDSCs [63]. On the other hand, the role of mitogen-activated protein kinase (MAPK) activation is known to be crucial in the regulation of pathways involved in the expansion of MDSCs [64]. The beneficial effects mediated by AMPK activation could therefore be induced by the inhibition of MDSCs functions, and the authors proposed that AMPK activators could be promising drug candidates for cancer therapy [64, 65].

In a murine model of neuroblastoma, the adoptive recipient leukocyte infusion (RLI) triggered a potent cytotoxic T cell response against the tumor. Conversely, the RLI also provoked a systemic expansion of MDSCs that weakened the CD8 anti-tumoral effect. When these MDSCs were depleted with 5-FU the inhibition of tumor growth and OS were improved significantly, suggesting that MDSCs targeting is a novel approach for increasing RLI effectiveness [66]. In another study, Eriksson et al*.* reported reduced levels of Tregs and PMN-MDSCs in pancreatic cancer patients treated with gemcitabine [67]. Weiss et al*.* demonstrated, in two murine models, the importance of targeting MDSCs and Tregs in anti-tumor immunotherapy. They described how this MDSCs and Treg reduction was Fas-dependent and, consistent with the conclusions of other authors, noted the importance of targeting these regulatory cells in order to ensure that the therapies produced successful anti-tumoral activity [68]. Dang et al*.* investigated the expression of TLR8 on MDSCs and the effect of motolimod, a TLR8 agonist, on MDSCs survival and function. They found that motolimod treatment reduced MDSCs levels in healthy donors and cancer patients, and concluded that TLR8 agonists could be deployed in conjunction with cancer immunotherapeutic approaches in order to enhance the anti-tumor effects of the adaptive immune response [69]. Davis et al*.* demonstrated functional inhibition of MDSCs with IPI-145, an inhibitor of PI3Kδ and PI3Kγ isoforms that enhances responses to PD-L1 blockade. These results offer a proof of concept for the low-dose use of isoform-specific PI3Kδ/γ inhibitors to suppress MDSCs and thereby enhance responses to the immune checkpoint blockade [70].

Other approaches focus on promoting MDSCs differentiation. A few years ago, Gabrilovich´s group demonstrated how all-trans-retinoic acid (ATRA) induces MDSCs differentiation into macrophages and dendritic cells (DCs) [71]. In 2018, Tobin et al*.* conducted a randomized phase II clinical trial in which they treated advanced melanoma patients with ipilimumab monotherapy, or ipilimumab plus ATRA. They showed that treatment with ATRA reduced MDSCs function in mixed lymphocyte reactions. ATRA also reduced the expression of immunosuppressive genes by MDSCs. Finally, ATRA significantly decreased the frequency of circulating MDSCs relative to ipilimumab treatment alone in advanced-stage melanoma patients [9].

Results published by Xin Lu et al. shed light on the field of metastatic castration-resistant prostate cancer (mCRPC) in relation to the use of immune checkpoint blockade for the purpose of blocking MDSCs. They developed a chimeric mouse model of mCRPC with which they demonstrated that combined therapies were capable of increasing IL-1ra and suppressing MDSCs-promoting cytokines. The authors combined PDL1 blockade and the kinase inhibitors cabozantinib and BEZ235, which induce a reduction in MDSCs function. This combined therapy proved to be more effective than single therapy [72].

These findings highlight the necessity of including MDSCs neutralization in novel strategies of combined cancer treatment.

The combination of immunoregulatory treatments with immune checkpoint blockade might represent a novel beneficial approach. The regulatory effects of diarylheptanoid curcumin on STAT3 and JAK2 signaling involved the decrease of IL-6 production by MDSCs but with no adverse effects detected [73]. Conversely, when STAT3 inhibitors were applied in clinical settings for targeting tumor-associated myeloid cells, the studies were disrupted by the side effects [74]. Additional approaches aimed at blocking STAT3 exploit the administration of siRNA and decoy oligonucleotides. For instance, AZD9150 is a STAT3 oligonucleotide inhibitor under investigation in combination with immune-checkpoint inhibitors in phase I/II clinical trials [10].

Various approaches that aim to target MDSCs have explored the blockade of MDSCs mobilization from the bone marrow. In this regard, bisphosphonates, drugs administered in cancer patients with bone metastases, have an MMP-9 inhibitory effect that is related to reduced MDSCs expansion in peripheral blood and bone marrow [75]. This reduction overcame the immune suppression and potentiated the anti-tumor response induced by immunization against the p185/HER-2. In spite of these results, most clinical trials of MMP inhibitors in recent decades have failed because of side effects: MMP regulates multiple signaling targets and the inhibition of some MMPs could have pro-tumorigenic effects that impair the benefits of target inhibition**.** These effects might be responsible for the failure of MMP inhibitors in clinical trials [76]. More recently, more specific MMP inhibitors with improved toxicity have been developed [77]. To date, several studies have indicated that modulating MMP activity can improve immunotherapy. As a consequence, several MMP inhibitors are the subject of clinic trials [62, 77].

More strategies aimed at decreasing the recruitment of MDSCs such as targeting tumor glutamine metabolism [78] and targeting chemokines [79] have been described.

Huber et al*.* described microRNAs associated with MDSCs features and shorter progression-free survival (PFS) and OS in melanoma patients treated with immune checkpoint inhibitors. These miRs were responsible for the conversion of monocytes into MDSCs mediated by melanoma extracellular vesicles (EVs) [80]. The authors argued that the role of the identified MDSC-miRs may reflect functional features that MDSCs display upon immune activation triggered by ICIs, because resistance to ICIs can be reverted by myeloid cell depletion. This highlights the need for drugs that are capable of blocking myeloid cells dysfunction. Hence, MDSC-related miRs are potential biomarkers for assessing systemic immunosuppression and immunotherapy outcomes in cancer patients [80].

Limagne et al*.* reported the outcomes of a prospective immunomonitoring study of 25 metastatic colorectal cancer (mCRC) patients treated with a first-line combination regimen of 5-fluorouracil 5-FU, oxaliplatin and bevacizumab (FOLFOX–bevacizumab), compared with healthy volunteers. FOLFOX–bevacizumab treatment produced a drop in gMDSCs in most patients and was associated with better survival. Moreover, those gMDSCs expressing higher levels of PD-L1, CD39 and CD73 showed potent immunosuppressive activity that could be reversed by blocking the CD39/CD73 and PD-1/PD-L1 axes [81].

The inhibition of prostaglandin E2 receptor 4 (EP4) in MDSCs may offer a strategy for enhancing the efficacy of immunotherapy in colorectal cancer [82]. *Li T *et al. showed that pharmaceutical inhibition of c-Rel in mice markedly inhibited cancer growth and suggest c-Rel as a myeloid checkpoint that may be targeted for treating cancer [83]. Similarly, S100A9-derived peptides conjugated to antibody Fc [84] and targeting the TNF-related apoptosis-induced ligand (TRAIL) receptor could be a potent and selective method for MDSCs depletion [85]. Several drugs against CSF1R have shown promising antitumor efficacies by inhibiting the survival of M-MDSCs and tumor associated macrophages (TAMs) and are being tested in in cancer patients [86].

In summary, several candidate drugs have been proposed for reducing MDSCs frequency and function; other approaches aim to modulate MDSCs differentiation or mobilization. A combination of immunoregulatory drugs with ICBs might be more effective in targeting MDSCs as anti-tumor immunotherapy.

### TAMs

Macrophages play an important role in regulating the innate immune system. On the one hand, they promote inflammation and eliminate pathogens. On the other, depending on their specific responses and cytokines, macrophages can induce immune stimulation and immunosuppression as well as promote or inhibit inflammation [87, 88]. Macrophages display a high degree of plasticity in response to different microenvironments [89]. Classic macrophages (M1) are known for their role in promoting immune responses. Conversely, M2 macrophages are associated with a reduction of tissue inflammation [90]. M2 macrophages can be classified into several populations based on their specific functions: M2a induced by Type2 cytokines, are responsible for mediating tissue repair; M2b induced by immune complexes, TLRs and IL-1R are known for their role in immunoregulation; M2c macrophages induced by anti-inflammatory cytokines affect phagocytosis and M2d induced by IL-6 like cytokines participate in angiogenesis [91].

**Fig. 4:**
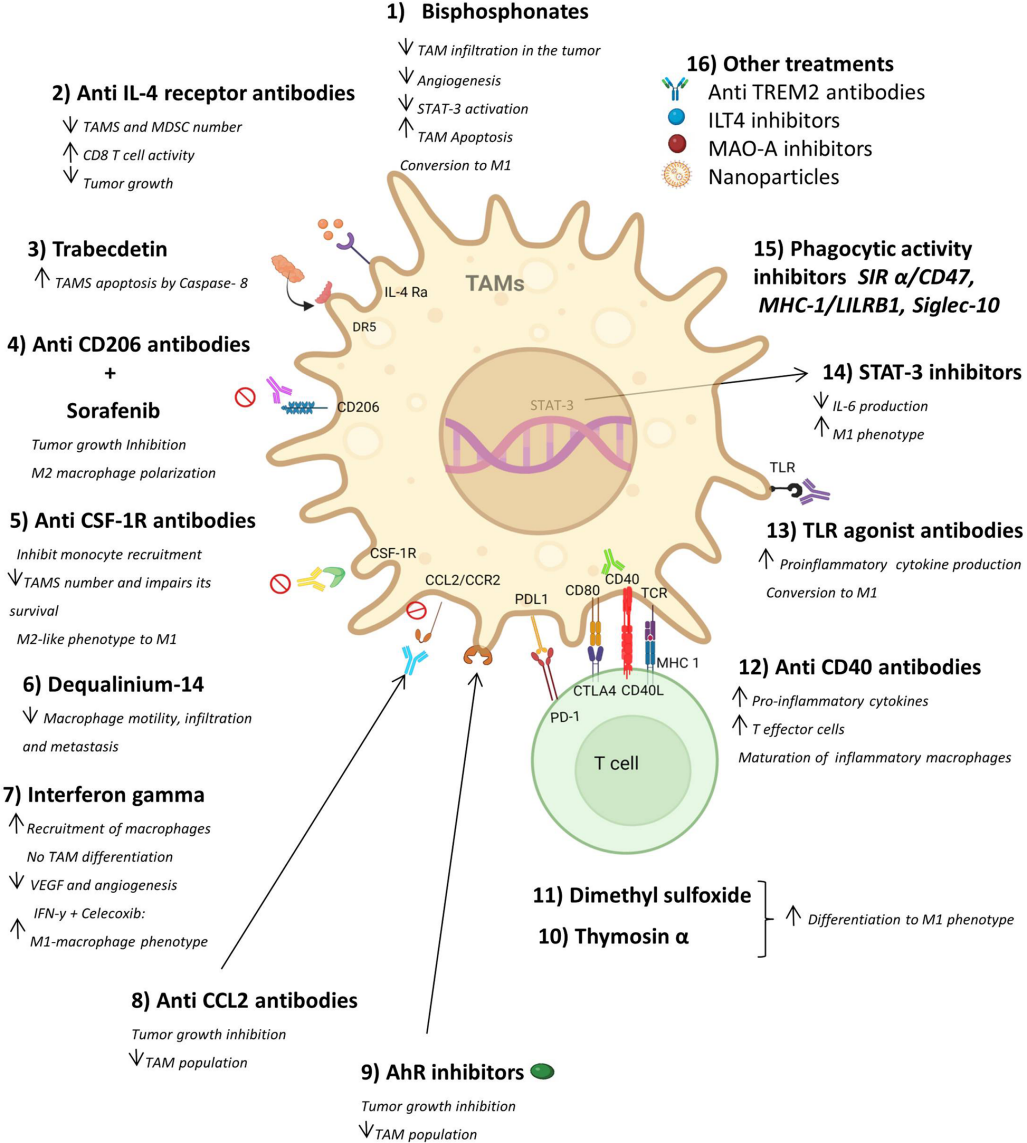
Effect of immunotherapy on TAMs. The figure displays treatments that aim to prevent the expansion of TAMs, consequently enhancing the anti-tumoral effect. The drugs work by promoting apoptosis (which reduces TAM frequencies)and TAM differentiation to M1 macrophages, and by blocking TAM mobilization, infiltration and metastasis. On the other hand, there is an increase in pro-inflammatory cytokine release and a reduction in the phagocytic activity of macrophages. Also, the use of these treatments combined with chemotherapy or other ICIs may be synergistic, bursting the immune response against the tumor. Created with Biorender.com

It is accepted that TAMs influence the tumorigenic process because they promote immunosuppression in the tumor microenvironment. In this regard, several studies have demonstrated that regulation of TAM responses may enhance immunotherapy [92]. Therefore, a wide range of strategies to deplete TAMs have been investigated in experimental settings and are now considered a promising therapeutic approach in the clinic [92] (Fig. 4).

Bisphosphonates have been successful in reducing TAM infiltration in vivo as they promote transformation of M2-like macrophages into M1-like macrophages. A large number of bisphosphonate derivatives are used to inhibit TAMs and treat tumors [93]. Likewise, an RNA aptamer that blocks the murine or human IL-4receptor-α (IL4Rα or CD124) can promote TAM elimination, an effect that is associated with an increased number of tumor-infiltrating T cells and a reduction in tumor growth [94]. Allavena et al. have shown that trabectedin activates caspase-8 and induces apoptosis in the presence of death receptor 5 (DR5), which is present in TAMs with death receptors [95].

In addition, specific antibodies can be used to reduce TAMs. Zhang et al*.* [96] synthesized IRD-αCD206, a TAM probe, that conjugated anti-CD206 antibodies with near-infrared phthalocyanine dye and successfully inhibited growth in a sorafenib-resistant tumor model. CD11b is an important molecule expressed in myeloid cells that works as an oncogene that can be targeted in colorectal cancer (CRC) [97]. Also, a recombinant immunotoxin consisting of FR-β monoclonal antibodies (mAbs), which are expressed in TAMs, suppressed tumor growth in a glioma model by depleting TAMs [98].

Other mechanisms targeting TAMs have been developed to inhibit monocyte recruitment. In this regard, the inhibition of CSF-1/CSF-1 receptor (CSF-1R) signaling has been effective because this axis is essential for macrophage survival. Also, targeting CSF-1/CSF-1R alters macrophage polarization and blocks glioma progression [99]. RG7155, a monoclonal antibody that inhibits CSF-1R, has been shown to inhibit CSF-1R and thereby decrease F4/80 + TAMs in vitro and in vivo [100]. Preclinical studies have demonstrated that another CSF-1R inhibitor, BLZ945, inhibits tumor growth in different mouse models [101] and its potential use in treating solid tumors is being studied in clinical trials [12].

The anti-tumor agent dequalinium-14 can reduce macrophage motility, inhibit macrophage infiltration of irradiated tumors, and reduce the extent of metastasis in locally irradiated mice. Interferon-γ (IFN-γ) may induce recruitment of monocytes/macrophages into the tumor microenvironment but inhibits their differentiation into TAMs in vivo; this effect may reduce the concentration of VEGF and angiogenesis in a tumor [102]. Anti-CCL2 antibodies have been shown to inhibit tumor growth in several animal models, such as glioma, colon cancer, prostate cancer, and melanoma. Tumor growth inhibition is observed with the use of Anti-CCL2 antibodies alone or in combination with chemotherapeutic drugs [13, 14]. In humans, a phase 1b clinical study showed that the combination of chemotherapy with the anti-CCL2 antibody, carlumab, for treatment of solid tumors was well tolerated but could sequester CCL2 for only a short time [13]. Inhibition of CCR2 can also block the CCL2/CCR2 axis to reduce the TAM population. PF-136309, a CCR2 inhibitor, proved to be safe and tolerable when used in combination with chemotherapy, yielding an objective tumor response in clinical trials [14].

A different approach involves regulating TAM polarization. For example, dimethyl sulfoxide can revert TAM polarization from M2 to M1 in a mouse 4T1 breast cancer model [103]. Overmeire et al*.* demonstrated that M-CSFR signaling shapes the M2-like phenotype in TAMs, and its blockade favored the shift towards M1-like TAMs [104]. IFN-γ and celecoxib increased the percentage of M1 macrophages and decreased that of M2 macrophages in lung tumors, suggesting that IFN-γ and celecoxib have the potential to be further optimized as a new anti-cancer therapy [105].

As mentioned above, the release of pro-inflammatory cytokines such as IFN-γ, TNF-α, and IL-12 induce transformation of TAMs into M1-like macrophages. Therefore, promoting the release of inflammatory factors, specifically through antibodies and small molecular agents, can promote the tumor immune response. A large number of studies have shown that the use of CD40 mAb can upregulate the level of proinflammatory factors and enhance the body's response to tumor cells by regulating the adaptive and inherent immune systems [106, 107]. In addition, the combination of anti-CD40 and anti-CSF-1R not only promotes the maturation and differentiation of inflammatory macrophages and DCs but also drives the effective initiation of effector T cells during cancer immunotherapy [108]. In addition to CD40 antibodies, other small molecules can enhance the secretion of pro-inflammatory cytokines. Thymosin-α activates and converts TAMs into pro-inflammatory cell subsets that produce IL-1, TNF-α, reactive oxygen species (ROS), and nitric oxide (NO). Furthermore, several clinical trials have confirmed that thymosin-α can prolong the survival time of patients with metastatic melanoma and advanced non-small-cell lung cancer (NSCLC) [109].

TLRs stimulation can lead to the expression and secretion of a variety of pro-inflammatory cytokines such as TNF-α, IL-12, and IL-1 [110]. Several studies proved that TLR agonists induce pro-inflammatory cytokines and reprogram macrophages [111, 112]. In addition to TLR agonists inducing cytokine release, stimulator of IFN gene (STING) agonists have also been shown to reprogram macrophages [113–115].

In tumor models of colon and pancreatic cancers, TAMs produce IL-6 by activating the STAT3 signaling pathway to promote the proliferation of colon tumor cells [116, 117]. Therefore, STAT3 inhibitors affect polarization of TAMs in tumor therapy. Several studies have shown that application of STAT3 inhibitors to several tumor models has therapeutic effects [118–120]. Some studies report the importance of the cellular metabolism in TAMs as an approach to abrogating the immunosuppressive effects of TAMs [121, 122].

The phagocytic activity of macrophages can be inhibited by blocking the interaction signals with cell surface proteins. Weissman et al*.* described three different pathways that inhibit phagocytosis: the signal regulatory protein alpha (SIRPα)/CD47 pathway, the major histocompatibility complex class I/leukocyte immunoglobulin (Ig)-like receptor subfamily B member 1 (*MHC-1*/*LILRB1*) pathway, and the CD24/sialic acid-binding Ig-like lectin 10 (*Siglec-10*) pathway [123–125].

Currently, since blocking those pathways enables phagocytosis, “don't eat me” signals are considered to be phagocytic checkpoints in macrophages, which serve as specific immune checkpoints for innate immunity. Some drugs targeting the SIRPα/CD47 signaling pathway have been widely studied in anti-tumor treatment, and most have been subjected to clinical trials [126, 127]. However, drugs targeting the *MHC-1*/*LILRB1* and *Siglec-10* pathways are still being investigated.

In a report published in 2021, the authors identified Fc domain-enhanced anti-TREM2 monoclonal antibody therapy promotes anti-tumor responses by modulation of TAM populations [128]. In non-small cell lung cancer (NSCLC) patients ILT4 inhibition prevented immunosuppression and tumor promotion by bloquing the recruitment of M2-like TAMs [129]. Monoamine oxidase A (MAO-A) inhibition treatment induces TAM reprogramming and suppresses tumor growth in preclinical mouse syngeneic and human xenograft tumor models [130]. Another recent report published by *Hezaveh K *et al*.* [131] demonstrated that pharmacologic inhibition of aryl hydrocarbon receptor (AhR) in myeloid cells reduced pancreatic ductal adenocarcinoma growth and improved efficacy of immune checkpoint blockade, and increased intra-tumoral frequencies of IFNγ^+^CD8^+^ T cells. The application of nanoparticles to target the tumor microenvironment is also being explored in combination with other therapies [132].

In conclusion, several strategies for depleting TAMs in the tumor microenvironment are being explored and are producing promising results. The use of these strategies combined with chemotherapy or immunotherapy could potentiate the anti-tumoral effect by promoting the immune response to the tumor.

### Other regulatory cells

The effect of immunotherapy in other cells with immunoregulatory properties, such as regulatory DC, B cells or MSCs, is still under investigation**.** Although there are very few reports about the role of Bregs in human cancer, some preclinical studies have targeted them in a variety of cancer models [133]. Mitogen/extracellular signal regulated kinase (MEK) is an intermediary component of the MAPK pathway, and its inhibition affects tumors in which MAPK is activated, alone or in combination with other therapies. Mechanistically, MEK inhibition may down regulate the expression of surface molecules associated with suppressive functions [134–136].

Das and colleagues investigated Bruton’s tyrosine kinase (BTK) as a potential modulator of Breg differentiation and the immunosuppressive function [137] and found that tirabrutinib, a BTK inhibitor, suppressed Breg differentiation as well as IL-10 and IL-35 production in vitro.

These studies have confirmed that the inhibition of Breg may help block cancer progression. Further research is needed to develop a Breg-targeting therapeutic regimen for cancer.

Though immunotherapy strategies, such as DC-based cancer vaccination, have been developed that are based on the ability of DCs to coordinate innate and adaptive immune responses [19], the role of TolDC, which is related to the effect of immunotherapy in cancer, remains poorly studied.

In the context of the tumor microenvironment, tumor cells can promote DCs with regulatory features [138]. Several approaches have been pursued that aim to activate DCs to suppress cancer progression, among them immunotherapy, which can affect DCs, promoting their differentiation functions.

The anti-tumoral immune response induced by DCs can be amplified using monoclonal antibodies against coinhibitory receptors (such as the PD1–PDL1 axis) or antibodies that potentiate the activation of costimulatory receptors (such as CD137) on T cells. Experimental melanomas with stabilized β-catenin signaling are associated with reduced cDC1 tumor infiltration and nonresponsiveness to immune checkpoint blockade (ICB) therapy. Indeed, vaccination with naturally occurring cDC1s loaded with immunogenic cell death-derived, whole-tumor antigen can synergize with anti-PD1 treatment [138].

Tumor antigen-loaded cDC1s were transferred into three cancer models in combination with anti-PD1 treatment, which had a strong synergistic effect [139]. Moreover, tumors grafted onto BATF3-deficient mice, which lack cDC1s, did not respond to anti-PD1, anti-PDL1, or anti-CD137 treatments, and SEC22B-mediated cross-presentation of TAAs by DCsis necessary for effective PD1 blockade therapy [140].

Synergy of TLR-mediated activation of DCs and ICB can be further improved by FLT3L-mediated expansion of DC populations [141].

Further evidence that cross-priming is the critical function mediated by cDC1 in this context has come from WDFY4-deficient mice, which fail to reject immunogenic tumors due to a defect in a vesicular transport pathway needed for cross-presentation [142].

*Wdfy4* ^−/−^ mice failed to prime virus-specific CD8^+^ T cells in vivo or induce tumor rejection, revealing a critical role for cross-presentation in anti-viral and anti-tumor immunity [142].

Enhancing DC functionality may improve and/or broaden responsiveness to ICB regimens. cGAS and STING are both necessary for intrinsic anti-tumor immunity and efficient responses to anti-PDL1, which is at least partially mediated by DCs [143].

Targeting type I interferons to activate cDC1s also improves anti-PDL1 treatment, suggesting that tumor DCs may require activation to support ICB-induced effector T cell activity.

Increasing DC chemokine production, may also increase responsiveness to ICB [144]. In turn, ICB promotes DC accumulation within the TME. Combining pembrolizumab (anti-PD1) treatment with TLR9 agonists is associated with an elevated tumor-infiltrating DC signature and an initial clinical benefit [145].

Mesenchymal stem cells (MSCs) are multipotent stromal cells that can differentiate into various cell types. A large number of studies have shown the beneficial effects of MSC-based therapies in treating various pathologies [146]. However, the therapeutic potential of MSCs in cancer is still controversial. Some studies indicate that they may contribute to cancer pathogenesis: MSCs can migrate to chronic inflammatory sites such as cancer, where they contribute to metastasis by secreting TGF, which promotes EMT [147]. MSCs inhibit the proliferation of T and B cells [148], suppress the activation of natural killer cells [149], and prevent generation and maturation of monocyte-derived dendritic cells [150]. Furthermore, MSCs can promote the generation of regulatory T cells [151], which exert immunosuppressive effects. However, the unmodified MSCs have been shown to have anti-tumor effects in vitro and in several mouse models of cancer [152].

The unique ability of MSCs to home to tumors and to directly transport anti-cancer agents to neoplastic niches renders them potential therapeutic vehicles for lung cancer. Genetic engineering is one of the most common strategies used to produce MSCs delivering tumor-suppressing agents into cancer cells. Several studies provide compelling evidence that MSCs can be genetically engineered to deliver anti-tumor drugs (PTX, DOX) and immunomodulatory factors (IL-12, IL-24, IFN-ϒ, IFN-β, TRAIL, PEDF, apotin, CDA/UPRT and CX3CL1) to target cells, thereby conferring anti-tumor/anti-metastatic actions [153].

Aside from their anti-cancer effects, MSCs are of special relevance for personalized cell-based therapies because they can be easily obtained by minimally invasive procedures and rapidly scaled up [154]. To date, 25 clinical trials that aim to use MSCs under various cancer conditions have been registered on ClinicalTrials.gov. Fourteen of these trials are using MSCs as a therapeutic agent to treat cancer directly. The use of MSCs as Trojan horses to deliver therapeutic factors represents an important step forward in the application of MSC-based therapies to provide more efficient cancer treatment [155].

The effect of immunotherapy in other cells with immunoregulatory properties, such as CD8 and NK regulatory cells remains to be determined. In this regard, in spite of the lack of studies regarding these regulatory cells in human cancer, it is likely that strategies developed to target other regulatory cells will also affect these subpopulations. 

## Conclusions

The extent of the immune infiltrate can be a good prognostic indicator in some cancers, but the anti-tumor immune response is, in most cases, insufficient to prevent disease progression. Several mechanisms and cell types are involved in negatively regulating the immune response, and the infiltration of large numbers of regulatory cells into tumor tissues is associated with poor prognosis.

In inflamed tumors, negative immune regulatory factors tend to dominate due to the chronic nature of the immune infiltrate. In cold tumors, it has been postulated that resistance to treatment may be due to low antigenicity, which causes less lymphocytic infiltration and is associated with an increase in the frequency of immunosuppressive cells and other factors.

Therapeutic success may depend on the phase and state of anti-tumor immune responses as the timing of the treatments seems to be critical to the differential control of these regulatory cells. When an immunosuppressive tumor microenvironment is already established, it will be more difficult to overcome and the immunotherapy will be less effective.

Although there are some current indications of the use of ICIs as first-line treatments, one of the main difficulties in clinical trials is that they were carried out in patients with advanced-stage tumors that were refractory to chemotherapy. Therefore, in tumors with low immunotherapeutic efficacy, it is plausible to consider that their failure to reach clinical end-points was due to the trial design.

Another challenge is that, in order to have validated drugs, it is necessary to study the possible anti-target effects of these molecules. The improvement in the design of clinical trials and the deep knowledge of the target and off-target effects of the treatments could lead to improved toxicity profiles and more selective drugs with less severe side-effects.

The discovery of new molecular targets could also help predict the tumors that are most immunogenic and most likely to respond to treatment, thereby making it possible to detect responders to immunotherapy.

In tumors characterized by a highly immunosuppressive environment, the development of new treatment options should certainly explore the efficacy of different drug combinations.

Numerous ongoing studies and clinical trials are exploring chemo-immunotherapeutic combinations that aim not only to eradicate the tumor mass but also to neutralize tumor-induced immunosuppression, thereby facilitating the effect of concurrent immunotherapy. Results obtained from these studies will shed light on the field and enable established approaches to be modified in order to better treat cancer patients in the next few years.

## Data Availability

Not applicable.

## Abbreviations

Tregs: Regulatory T cells
Mregs: Regulatory macrophages
MDSCs: Myeloid suppressor cells
tolDCs: Tolerogenic dendritic cells
Bregs: Regulatory B cells
MSCs: Mesenchymal stem cells
ICIs: immune checkpoint inhibitors
CTLA-4: cytotoxic T-lymphocyte antigen 4
PD-1: programmed cell death protein 1
PBMCs: peripheral blood mononuclear cells
TILs: tumor-infiltrating lymphocytes
mAbs: monoclonal antibodies
ATG: anti-thymocyte globulin
MAPK: mitogen-activated protein kinase
AMPK: mitogen-activated protein kinase pathway
RLI: recipient leukocyte infusion
TAMs: tumor-associated macrophages
